# Early Montessori education shows delayed benefits for mathematical problem-solving in a 5-year longitudinal randomized controlled trial

**DOI:** 10.1038/s41598-025-27687-2

**Published:** 2025-12-17

**Authors:** Sarah Le Diagon, Jean-Baptiste Van der Henst, Jérôme Prado

**Affiliations:** Centre de Recherche en Neurosciences de Lyon (CRNL), INSERM U1028, CNRS UMR5292, Université de Lyon, Bron, France

**Keywords:** Montessori, Early childhood education, Academic achievement, Longitudinal design, Randomized controlled study, Education, Psychology, Psychology

## Abstract

Early childhood education may have the potential to narrow the income achievement gap. However, studies have shown substantial intervention fadeout, calling for rigorous assessment of the long-term effects of early childhood programs. In a randomized controlled study, Courtier et al. (2021) found that an adapted Montessori curriculum led to larger reading gains in kindergarten than conventional public education in France. Participants from that intervention were recovered five years later while in conventional classrooms and once again tested on academic, cognitive, and social skills (*N* = 97; M_age_=10–11). Children who benefited from the adapted Montessori curriculum no longer showed better reading skills than their peers (d=-0.07). However, they outperformed their peers on math problem-solving (d = 0.58), an effect not present in kindergarten.

**Supplementary Information:**

The online version contains supplementary material available at 10.1038/s41598-025-27687-2.

Early childhood interventions are often considered an effective public investment for reducing later socioeconomic inequalities in educational achievements^1^. This idea has been supported by landmark randomized controlled studies showing long-term positive impacts of preschool programs^2–4^. However, evaluations of more recent programs have provided inconsistent evidence for long-term effects of early education interventions^5^, with frequent fade-out of immediate benefits over time^6–8^. This calls into question the generalization of effects from early randomized controlled interventions, which were notably limited by a lack of access to childcare and education for control-group counterparts^5^. Thus, the estimated impacts might reflect the absence of educational opportunities for the control groups rather than the intrinsic effectiveness of the programmes. Given the importance of early care and education in current public discourse, it remains crucial to rigorously measure the long-term effects of specific early childhood education programs by comparing them to high-quality alternatives.

An early childhood education program that has been gaining popularity in recent years is Montessori education. The Montessori early childhood curriculum (which is the first part of a curriculum that can go up to high-school) typically includes two years of preschool and one year of kindergarten, catering to children aged 3 to 6 years old. Montessori early childhood education differs from other pedagogical approaches with respect to the classroom environment, the role of children, and the role of teachers^9^. For example, classrooms span three-year age groupings (i.e., 3 to 6 years old), with a space organized according to different domains of the curriculum (e.g., math, language, art, practical life)^10^. The Montessori method also relies on manipulable, multi-sensory materials that are self-correcting^11^. It places a strong emphasis on self-learning, with children being encouraged to freely choose activities from those available and decide how long to spend on them. Work periods are relatively long and uninterrupted^10^. Finally, teachers serve as guides for children’s learning more than they directly teach content as is often the case in conventional education^10^.

Another feature of Montessori early childhood education is its didactic content. In the language domain, for example, the curriculum combines multi-sensory activities with a phonetic approach to reading acquisition where children systematically learn letter-sound correspondences before spelling or decoding words. In the math domain, children are introduced to various concepts through manipulatives. Emphasis is placed on the relation between quantities and numerical symbols, with children being introduced to the base-10 system, as well as arithmetic and fractions. Finally, the curriculum is full of activities promoting coordination and concentration, making fine distinctions across several sense bases, and emphasizing cultural and scientific awareness. Interestingly, Montessori education has often received support from cognitive psychologists for several reasons. First, its sensory-motor focus resonates with embodied cognition, which suggests that learning is grounded in physical interaction with the environment^12,13^. Second, the emphasis of Montessori education on self-directed activity, concentration, and deliberate practice may support the development of executive functions^14^, which have been shown to be associated with academic achievement^15^. Third, open and mixed-age classrooms might promote collaboration, peer learning, and socio-emotional skills that relate to academic outcomes^16,17^. Finally, its approach to early literacy and numeracy aligns with evidence from cognitive science regarding effective instructional practices^11,18^.

The actual impact of Montessori education on children’s development and skills has been evaluated by a body of literature over the past few decades, recently synthesized in two meta-analyses^19,20^. Both meta-analyses found an advantage of Montessori pedagogy compared to the counterfactual for domains such as cognitive abilities, creativity, motor skills, and academic achievement (i.e., language and math), with moderate to large effect sizes.

The long-term benefits of Montessori early childhood education, however, remain unclear. There are four reasons for this state of affairs. First, overall estimates from the meta-analyses above include studies conducted from preschool to high school, and therefore do not necessarily reflect an influence of early childhood education. For instance, Randolph et al. (2023) suggests that overall benefits from Montessori education are strongest during elementary school years^20^.

Second, very few studies have used a design in which the assignment of children to Montessori education was randomized. This is problematic because children whose parents have chosen Montessori education might differ from other children in systematic ways. For example, families who seek Montessori placement may have higher socioeconomic status, greater educational resources, and stronger investment in alternative pedagogies, which are factors that may predict academic outcomes. These children may also differ with respect to academic, cognitive, and socioemotional skills at baseline (which are often not controlled^20^). The few studies that have used randomization have also often relied on lottery-based designs^21,22^, in which families apply for admission to oversubscribed Montessori programs and children are randomly selected via lottery among applicants. Although lottery-based designs minimize bias between groups of children who have won versus lost the lottery (both groups have parents motivated to seek Montessori education), they might lack external validity as they exclusively focus on children of parents who have an interest in the program and may not generalize to families who would not actively seek Montessori placement. Thus, non-lottery studies using gold standard randomized-controlled designs remain needed to best estimate the effect of Montessori education.

Third, the large majority of studies have focused on the immediate effects of Montessori education, measuring children’s outcomes only at the end of the intervention^19,20^. This lack of longitudinal evidence is problematic because intervention studies in general tend to show fade-out of immediate benefits over time^7^. The studies that have followed children longitudinally several years after completing Montessori preschool or kindergarten do suggest some long-term academic benefits^23–27^. However, these studies have some flaws that make their findings difficult to interpret, including low sample sizes and low fidelity of implementation of the Montessori curriculum^23,26,28,29^ as well as lack of randomized control groups^23,24,30,31^.

Finally, any evaluation of the potential benefits of Montessori early childhood education hinges on a comparison with a counterfactual group. The characteristics of this group, however, greatly vary between studies and may often include preschool programs that are play-based and much less structured than Montessori education^20^. This makes it difficult to attribute effects to the Montessori curriculum itself, as differences may also stem from the reliance on a more structured program (versus a less structured program).

In a recent study, Courtier and colleagues (2021) were able to take advantage of a unique experimental context in which a Montessori-inspired early childhood curriculum was implemented in some classrooms of a public school serving disadvantaged children in France^32^. What made the setting unique is that children were randomly assigned either to Montessori classrooms or to conventional classrooms, without parents having explicitly chosen Montessori as in lottery-based designs. Investigating the effects of Montessori education in this context minimizes not only selection bias but also the generalizability limitations inherent in lottery-based studies, which exclusively sample families motivated to seek Montessori education. Montessori classrooms followed an adaptation of the Montessori curriculum, which consisted of fewer materials, shorter work periods, and relatively limited Montessori teacher training. An objective scale nonetheless ensured that these classrooms were relatively comparable to classrooms from a school accredited by the French Montessori association, both in terms of general characteristics and time spent by children engaged in Montessori activities (see extensive details on the curriculum in Courtier et al., 2021)^32^. Conventional classrooms, in contrast, followed the French public curriculum, a structured teacher-centered curriculum aimed at developing socio-emotional skills as well as early literacy and numeracy. Though children were tested on a range of math, language, executive function, and social skills in kindergarten, the results only showed a significant advantage in reading skills for children who followed the Montessori pedagogy compared to the conventional group.

These findings raise the question of whether children who followed the three-year Montessori-inspired early childhood curriculum still show a reading advantage several years later (i.e., fifth grade). Testing this specific hypothesis about reading skills, which was preregistered, represents our primary confirmatory analysis. We also conducted exploratory analyses to examine potential late-appearing benefits in other skills, investigating whether children who followed the Montessori early childhood curriculum show advantages that were not present at the end of kindergarten (i.e., a so-called *sleeper effect*). To test these confirmatory and exploratory hypotheses, we recovered most children from the Courtier et al. (2021) study five years later, while they were in 5th grade in two public elementary schools. We then tested their academic, cognitive, and social skills, using a battery of age-appropriate tests (see Supplementary Table S1).

## Methods

### Participants

Courtier et al. (2021) tested an initial sample of 150 children from a public “école maternelle” in France (the French preschool system), yielding a final sample of 131 children^32^. Children either followed the Montessori early childhood curriculum or the French public preschool and kindergarten curriculum. Five years later, we were able to locate 101 children from this initial sample, who either still attended the same school (school A, *n* = 73) or attended another school in the same neighborhood (school B, *n* = 28) as is standard in the French preschool and elementary school system. That neighborhood was a socioeconomically disadvantaged part of the greater Lyon area in France. As a result, family socioeconomic status of children was low (see Supplementary Information, SI). Data, which were from three cohorts, were collected in these two schools at the end of 5th grade (March 2022, March 2023, and March 2024). The study received ethical approval from the INSERM institutional review board (22–881). We confirm that all methods were performed in accordance with the relevant guidelines and regulations. Parents gave their written informed consent and children gave their assent to participate in the experiment.

Children of school staff (*n* = 1) and children with documented disabilities requiring specific classroom placement (*n* = 3) were exempt from the randomization and were therefore excluded from the analyses. Therefore, our final sample included 97 5th-graders. Thirty-nine children from the final sample were in the *Montessori group* (17 girls; 26 children in school A; M_age_=10.77 years old, SD = 0.30). All of these children had been randomly assigned to a classroom that followed the Montessori early childhood curriculum either when they first enrolled into public preschool at age 3 (*n* = 29) or later at age 4 (*n* = 5) or age 5 (*n* = 5). Random assignment to classrooms was performed by the school administration following standard French public-school procedures. This process involves complete randomization of classroom assignments and does not allow parents to request specific classroom placements. The 58 remaining children were in the *Conventional group*. These children had been randomly assigned to a classroom that followed the conventional French public preschool and kindergarten curriculum (27 girls; 43 children in school A; M_age_=10.73 years old, SD = 0.32). No children transferred from a Montessori classroom to a Conventional classroom during the 3 years of the curriculum. The 10 children who started the Montessori early childhood curriculum at age 4 and 5 had transferred from the conventional pedagogy. Supplementary Table S2 presents key sample characteristics at pre-K (age 4) for the participants on which follow-up data was available. There was baseline equivalence on all measures between Montessori and Conventional groups. A full description of the Montessori and conventional pedagogies that children followed in preschool and kindergarten is given in SI with additional details provided in Courtier et al. (2021)^32^. Supplementary Table S3 presents all of the end of treatment effects (i.e., scores at the end of kindergarten) for the participants on which follow-up data is available. These data replicate the findings from Courtier et al. (2021): the only difference between the groups across all measures was in terms of reading scores^32^.

From 1st to 5th grade, all children followed the same conventional French public elementary school curriculum (see SI for details about that curriculum). Attrition analyses revealed no significant difference in dropout rates between the Montessori (39.13%, 27/69) and Conventional groups (27.16%, 22/81) (χ²(1, 150) = 2.43, *p* = .119). Furthermore, participants who were not included in the follow-up did not differ from those who were in terms of vocabulary, reading, phonological awareness, math problem-solving and executive functions at pre-K (all ps > 0.272, all BF_01_ > 3.28). There was also no difference in the proportion of children from the Montessori group compared to the Conventional group between the two elementary schools (χ²(1, 97) = 0.63, *p* = .426).

### Preregistration

This study is part of a larger preregistered project examining longitudinal relations between early childhood skills and later academic outcomes (https://osf.io/8f6tb). The preregistration focused broadly on how early academic skills predict later outcomes, with our Montessori versus conventional curriculum comparison being one component of the larger design. Nonetheless, our preregistration specifies the measures and dependent variables used in 5th grade, describes the sample (and notably the fact that some children followed a 3-year Montessori curriculum whereas others followed a conventional curriculum), and states the basic analytical approach comparing the groups using frequentist and Bayesian tests. The preregistration also specifies that we expected higher reading scores for the Montessori than the Conventional group in 5th grade. There were two main deviations from the preregistration with respect to the comparison between Montessori and Conventional groups. First, while our preregistration used the term “three years of kindergarten” to describe the 3-year curriculum of the French “école maternelle”, we refer to that curriculum as two years of preschool and one year of kindergarten in the current paper as it is more comparable to the US system. Second, although our preregistered measures included questionnaires regarding bullying and well-being at school, these were designed for the broader project and were not included in the present analyses.

## Materials and procedure

Children were tested individually in a quiet room of their school for one hour and collectively with the whole class for another hour (see Supplementary Table S1 for when each test was administered). The experimenters were blind to the pedagogy followed by children in preschool and kindergarten. Children were assessed on their academic skills (reading fluency, math problem-solving, and arithmetic fluency), cognitive skills (short-term memory and working memory) and social skills (sharing and theory of mind). All tests, which were administered using paper and pencil, are described in Supplementary Table S1.

### Data analysis

Data were analyzed with Jamovi (v2.3.28) and R (v4.3.0). Test scores were entered in frequentist and Bayesian independent t-tests comparing the Montessori to the Conventional group. For frequentist analyses, p values were considered significant at a Bonferroni-corrected significance threshold of *p* = .006 (i.e., 0.05/8 measures). For Bayesian analyses, Bayes factor (BF) assessed the probability of the data under the null hypothesis (i.e., the absence of a difference between the two groups) and the probability of the data under the alternative hypothesis (i.e., the presence of a difference between the two groups). We used the default non-informative Cauchy distribution prior centered on zero with a width parameter of 0.707 (an effect size of 0 was used as a prior). BFs were interpreted according to guidelines by Jeffreys (1961)^33^. Given the randomized controlled design and baseline equivalence between groups on demographic characteristics and pretest measures (see **Supplementary Table S2**), our primary analyses did not include covariates. However, complementary analyses explore the effect of different covariates (math problem-solving performance in pre-K, vocabulary in pre-K, reading performance in pre-K, age at testing and sex), as well as more generally the robustness of our findings (see SI).

## Results

**Fig. 1:**
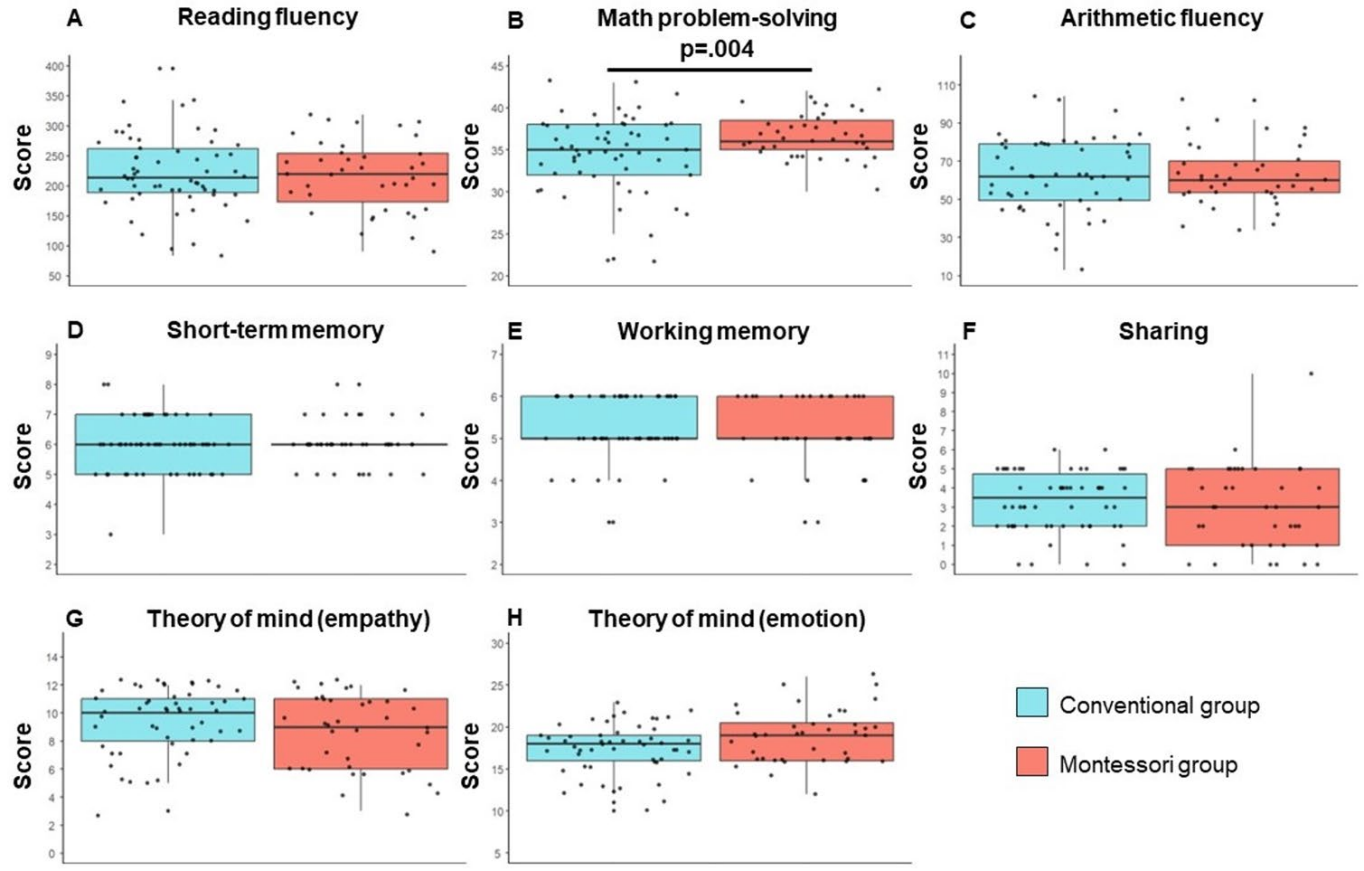
Box plots of scores for all tests as a function of the group. Each black dot represents a child. Thick horizontal black line within each box plot represents the median. Upper and bottom lines are the 25th (bottom) and 75th (upper) percentiles of each group’s distribution of values.

Scores by test and group are shown on Fig. 1 (see Supplementary Table S4 for exact mean performance). Results from both frequentist and Bayesian analyses are shown in Table 1. Contrary to our prediction (confirmatory hypothesis), there was no significant difference in reading fluency between the groups in 5th grade (*p* = .741), and Bayesian analyses indicated substantial evidence for a lack of difference (BF_01_ = 4.38) (see Table 1; Fig. 1A). However, exploratory analyses indicated that a significant difference between the Montessori and the Conventional groups was observed for math problem-solving (*p* = .004), such that children from the Montessori group had higher scores in 5th grade than children from the Conventional group (see Table 1; Fig. 1B). The size of that difference (d = 0.58) was very large as far as educational interventions are concerned^34^. Bayesian analyses also indicated substantial evidence for a difference between the groups in math problem-solving scores (BF_10_ = 4.77). None of the other exploratory comparisons were significant, with substantial evidence that scores did not differ between groups in tests of arithmetic fluency, short-term memory, working memory, and sharing, and anecdotal evidence for a difference in theory of mind (see Table 1; Fig. 1C and H). As detailed in SI, a series of complementary analyses demonstrated that our findings remain robust to the exclusion of outliers, inclusion of covariates, and exclusion of the few children with an incomplete Montessori early childhood curriculum (see Supplementary Table S5).

**Table 1:** Frequentist and bayesian independent sample t-tests.

	t	df	*p*	Cohen’s d	BF_10_	BF_01_
Reading fluency	-0.33	95	0.741	-0.07	0.23	**4.38**
Math problem-solving	2.94^a^	93.53	**0.004**	0.58	**4.77**	0.21
Arithmetic fluency	-0.14	88	0.892	-0.03	0.22	**4.46**
Short-term memory	0.15	95	0.879	0.03	0.22	**4.55**
Working memory	-0.58	95	0.561	-0.12	0.25	**3.96**
Sharing	-0.62^a^	63.15	0.540	-0.13	0.26	**3.80**
Theory of mind (empathy)	-1.41	89	0.162	-0.30	0.53	1.89
Theory of mind (emotion)	2.42	89	0.018	0.51	2.78	0.36

^a^A Welsh correction was applied for this test because the condition of equality of variances was violated. For frequentist statistics, the Bonferroni-corrected significance threshold was *p* < .006. Significant results are in bold. Cohen’s ds represent effect sizes that can be considered small (0.05), medium (0.05 to < 0.20) or large (≥ 0.20) in the context of an education intervention ^34^. Degrees of freedom (df) vary because some children were absent for some of the tests. Bayesian statistics: BFs > 3, which correspond to at least substantial evidence for either the null (BF_01_) or the alternative (BF_10_) hypothesis, are in bold.

## Discussion

Contrary to our prediction, we found substantial evidence for a lack of difference between the Montessori and the Conventional groups in terms of reading in 5th grade, suggesting an important fade-out of the Montessori advantage previously found in kindergarten^32^. In contrast, exploratory analyses indicated that 5th-graders from the Montessori group showed a relatively large advantage in terms of math problem-solving skills compared to other children, five years after the intervention.

Courtier et al. (2021) attributed the reading advantage observed for Montessori education at the end of kindergarten to a stronger emphasis on synthetic phonics, word production, and sensory-motor interactions than in the French public early childhood curriculum^32^. It is possible that the reading advantage observed in kindergarten faded because, beyond a certain point in learning to read, success is no longer hierarchically dependent on the early learning promoted by the Montessori pedagogy. However, it is also possible that the fade out is due to overlap between the literacy content taught in Montessori classrooms and in subsequent elementary school instruction. Indeed, when there is overlap between content taught during an early intervention and subsequent educational environments, children who did not receive the intervention initially will show steeper learning curves when they first encounter this content, leading to catch-up effects^35^. For example, all children followed the French elementary school curriculum after kindergarten. The curriculum makes use of both synthetic and analytic phonics in 1st grade, such that reading instruction may have repeated activities Montessori children had already mastered in earlier grades. This would have allowed children from the conventional group to catch up, while providing limited opportunities for Montessori children to build upon their early literacy advantages.

In contrast to reading, we did observe a large difference between the groups with respect to math problem-solving in 5th grade. Overall, these results appear consistent with findings from previous studies that also suggest an advantage for Montessori children in math compared to control groups several years after the end of the intervention, even when children no longer follow the Montessori pedagogy^24,28,36^. For example, Dohrmann and colleagues (2007) found that children who attended Montessori programs from preschool through 5th grade showed an advantage of approximately one-third of a standard deviation on standardized math and science tests in high school compared to demographically matched peers who attended traditional schools^24^. It is worth noting that the difference observed in the present paper emerged only for the math measure involving conceptual understanding, as Montessori and non-Montessori children performed similarly on quick calculation tasks. Critically, the exact same math problem-solving measure did not show a group difference at the end of kindergarten. There are a number of possibilities to explain such a so-called *sleeper effect*, which has been previously suggested in the context of Montessori education^20^.

First, Randolph and colleagues (2023) speculated that the outcomes of philosophical elements of Montessori early childhood education (e.g., free choice, classroom organization) may only be observed in the long run^20^. However, if broad pedagogical principles were the primary mechanism driving the observed effect, we might expect to see more widespread benefits across multiple measures. Instead, the specificity of the effect to math might point more strongly toward domain-specific aspects of the Montessori curriculum (see below).

Second, it is possible that the effects of early interventions on math skills may strengthen over time due to the hierarchical nature of mathematical learning. Though such sleeper effects remain relatively rare compared to fade-out, long-term benefits from early numeracy interventions are at least supported by some prior studies^8,37^. For example, Berkowitz and colleagues (2015) demonstrated that early math engagement predicted mathematics achievement a year later^37^. The emergence of this math advantage is particularly noteworthy given that children from the Montessori and Conventional groups showed no difference in quantitative knowledge at the end of kindergarten (see Supplementary Table S3). This pattern (where benefits emerge years after the intervention rather than appearing immediately or fading out) may represent a genuine sleeper effect that cannot be explained by simple cumulative advantage from early differences.

Finally, it is possible that the emergence of a group difference in 5th grade (where none existed in kindergarten) may in part come from the structure of the measure itself. Indeed, while early items of the Applied Problems subtest involve counting and basic arithmetic, subsequent items involve clock reading, currency calculations, and word problems that increasingly require understanding of base-10 and place-value. The Montessori early numeracy curriculum puts more emphasis on these concepts, and particularly understanding of base-10 and place-value, than the conventional French public curriculum in early grades (e.g., golden beads, which are small bead units grouped into tens, hundreds, and thousands, and Seguin boards are typical materials developing base-10 understanding). Therefore, a difference between groups might emerge in the long run because the measure itself more heavily relies on such skills in 5th grade than in kindergarten. This is for example consistent with studies suggesting that children from Montessori schools may have better understanding of base-10 than other children in kindergarten^38^ and later grades^39^. It is also possible that a particularly beneficial aspect of the Montessori curriculum with respect to math learning is its extensive use of manipulatives, which may help scaffold the concrete understanding of early math concepts and support later learning^40,41^.

The specificity of our findings regarding math problem-solving skills also warrants discussion. First, the advantage in math problem-solving but not arithmetic fluency suggests that long-term benefits of Montessori education may relate to conceptual understanding more than procedural efficiency. Both groups received extensive arithmetic practice in elementary school, which might have equalized arithmetic fluency, while Montessori’s emphasis on base-10 understanding may have provided conceptual foundations that become advantageous as problems increase in complexity. Second, the absence of long-term effects on executive functions and social skills is consistent with the lack of group differences on these measures at the end of kindergarten in our previous study^32^. While Montessori education theoretically emphasizes self-regulation and social development through mixed-age classrooms and self-directed learning, our findings do not provide evidence that this translates into measurable advantages in executive functions or social skills, either immediately or long-term. Therefore, observable benefits of early Montessori education may be more domain-specific than domain-general.

Several methodological considerations warrant discussion when interpreting our findings. While our study benefits from a randomized controlled design and a comprehensive battery of measures spanning multiple skills, we acknowledge that our sample size (*n* = 97) and attrition rate (32.66%) may impact the precision of our effect estimates. Effect sizes from educational interventions with smaller samples tend to have wider confidence intervals and may be susceptible to magnitude overestimation^34^. The observed effect size for math problem-solving (d = 0.58) is relatively large for an educational intervention measured several years post-treatment. While this could reflect a genuine sleeper effect of Montessori education on math skills, the combination of sample size constraints and multiple outcome measures raises the possibility that this single significant finding could have occurred by chance. However, we note that several factors argue against this interpretation, notably the fact that the effect survived our conservative Bonferroni correction (*p* = .004 vs. threshold of 0.006), is supported by Bayesian analyses (BF₁₀=4.77), and aligns theoretically with Montessori’s documented emphasis on base-10 understanding. Conversely, our sample size may have limited our ability to detect smaller but meaningful effects in other domains. For example, there was only anecdotal evidence for a potential advantage for the Montessori group in emotion recognition (see Table 1), despite past evidence that children who experience the Montessori pedagogy may show differences in emotion processing compared to other children^42^. It is possible that our study may have been underpowered to detect these effects. In general, replications with larger samples are needed not only to obtain more precise effect size estimates for the sleeper effect detected, but also to better characterize potential effects in other cognitive and social domains such as emotion recognition.

In sum, we demonstrate the fadeout and emergence of benefits from an early childhood Montessori intervention in 5th grade, using a randomized controlled design and a control group having received a qualitative early childhood curriculum. This experimental setting makes the study relatively unique in the literature. Together with Courtier et al., (2021), the present findings demonstrate the potential of Montessori early childhood education to promote long-term numeracy (see also^24^), but also show that early gains in literacy may only benefit children if elementary schools build on those gains.

## Supplementary Information

Below is the link to the electronic supplementary material.

Supplementary Material 1

## Data Availability

The data that support the present findings are available via the OSF at [https://osf.io/x5cna/files/wz6g4].
