# Supplementary material for: Early Montessori education shows delayed benefits for mathematical problem-solving in a 5-year longitudinal randomized controlled trial
Source: Sci Rep. 2025 Dec 17;15:43961. doi: 10.1038/s41598-025-27687-2 (PMC12711950; doi:10.1038/s41598-025-27687-2)
Supplement: Supplementary file 1 — Supplementary Material 1 [file 41598_2025_27687_MOESM1_ESM.pdf]

Supplementary Information

Socioeconomic background of participants

Both elementary schools are located in the same neighborhood of the Lyon suburban area. These schools are cost-free and largely cater to underprivileged children. At the time of the early childhood intervention, parents were asked to fill out a questionnaire about the home environment, including household income, number of siblings and number of languages heard at home. Though only a subset of the parents returned the questionnaire (n=51), yearly household income of parents was on average of €27,000, which was lower than the French median household income of €30,260 euros in 2018 (https://www.insee.fr/fr/statistiques/5371205?sommaire=5371304). As reported in Courtier et al. (2021), there was no difference in household income between the Conventional and the Montessori groups (BF_{01}= 3.23, t(49)= 0.18, p= .86). There was also no difference in number of siblings between the Conventional and the Montessori groups (BF_{01}= 1.23, t(50)= 1.58, p= .12). Note that data on participant ethnicity were not collected because the collection of such data is in principle illegal in France and would require an exceptional waiver from government agencies that they did not seek.

The relatively low socioeconomic status of children was also suggested by the official "social positioning index" (IPS) of the schools. The IPS is a measure provided by the French government that integrates various socioeconomic indicators such as educational attainment, occupational categories, and income levels of parents to assess the family socio-economic status of children in all French schools (https://www.data.gouv.fr/en/datasets/indices-de-position-sociale-dans-les-ecoles-de-france-metropolitaine-et-drom-version-2-1/). The IPS of both schools were among the 13.5% lowest IPS of the Lyon area, indicating that the schools catered to children from low socioeconomic backgrounds.

Criteria for exclusion of participants

Among the children we were able to locate, 4 children were excluded because their placement into Montessori or Conventional classrooms was not randomized (either because they were related to the staff, n = 1, or because they had a diagnostic of disability, n = 3).

Description of the adapted Montessori early childhood curriculum

As detailed in Courtier et al. (2021), children from the Montessori group followed an adaptation of the Montessori curriculum in preschool and kindergarten. Each Montessori classroom had one teacher and one teaching assistant for approximatively 30 children. Each classroom was mixed-age, with children from 3 to 6 years old. Thus, there were approximately 10 kindergarteners in each classroom each year. Children had 90 to 120 minutes of autonomous and free-choice work every half day. During those work periods, children chose their activities from the materials (appropriate for their learning level) displayed in the classroom. The materials were specifically designed (by Maria Montessori and collaborators) to teach specific skills through multi-sensoriality and auto-correction. Students could also choose the amount of time they worked on each material, where they sat to use the materials (around tables or on carpets on the floor), and if they wanted to work alone or with their peers. There was only one instance of each material in the classroom, so children were required to wait to work on a material that was already used by another child. The teacher's main role was to present new materials to children individually and to assist them when they deemed it necessary. They also observed children to evaluate their progression. There was no recess because children could choose to take breaks whenever they wanted. There was also a gymnastic class every morning before lunchtime.

With the exception of one teacher who completed an official training program from the Association Montessori Internationale (AMI) at the end of the second year of data collection,

teachers were largely self-trained in Montessori education. Their self-training consisted of reading Maria Montessori's books, interacting with several AMI-trained teachers from the Lyon area, observing classrooms from AMI-affiliated preschools and organizing weekly team meetings to share information among themselves. They also made extensive use of online resources. Teachers also participated in a two-day workshop organized by the official training institute of the French AMI affiliate. The goal of that workshop was to introduce the Montessori approach, focusing on children from 3 to 6. Finally, when one teacher was able to get funding to complete the official AMI training, this teacher had weekly meetings with the other teachers to pass on what he had learned.

The fidelity of implementation of the Montessori curriculum in classrooms was assessed by a scale available at https://osf.io/wqxkm. The scale assessed the fidelity of implementation with respect to three domains: the proportion of Montessori materials present in the classroom, the fundamental characteristics of Montessori education (e.g., organization of classrooms, mixed-age classrooms, presence of non-Montessori materials), and the proportion of children in the classroom engaged in a Montessori activity at one time point of the day. Scores on that scale for the Montessori classrooms were compared to a benchmark, which was the scores obtained on that same scale by classrooms from a private Montessori school accredited by the French affiliate of the AMI. As can be seen in Courtier et al. (2021), the classrooms that followed the adapted Montessori curriculum were faithful to the standard Montessori early childhood curriculum used in the AMI-accredited school in terms of both Montessori characteristics and activities. However, Montessori materials were less numerous in these classrooms than in AMI-accredited classrooms (a complete list of the materials present in the classrooms is given in Appendix S2 of Courtier et al., 2021). Finally, there were two main differences between the adapted Montessori curriculum and the standard Montessori curriculum. First, daily work periods were shorter (1.5–2 hr vs. 2.5 hr, respectively). Second,

most of the teachers held a degree from a conventional teacher's college and did not receive official AMI training. Therefore, Montessori classrooms were not as faithful to the standard Montessori early childhood curriculum as AMI-accredited classrooms, though they were still far more faithful to Montessori education than conventional classrooms (see below).

Description of the French conventional public preschool and kindergarten curriculum

Children from the conventional group followed the French conventional public curriculum in preschool and kindergarten (https://eduscol.education.fr/document/20062/download). Each classroom had one teacher and one teaching assistant for approximately 30 children. Classrooms were composed of either children from the same age group or children from two age groups (depending on the number of children registered for the school year). Conventional classrooms schedule was teacher-directed and highly structured. Each day usually included two to three whole class gatherings, one free play time at the beginning of the day, two 30-minutes learning activity time, one or two recess time and one gymnastics lesson. For learning activities, children were divided into small groups, with one group supervised by the teacher, one group supervised by the assistant and the other groups working autonomously on activities chosen by the teacher. Teachers were required to prepare the content of learning activities in accordance with the official French curriculum. This official curriculum includes five learning domains: language and literacy, physical well-being and motor development, art, numeracy, and general knowledge. The curriculum also defines the skills that should be (at a minimum) acquired by children at the end of kindergarten. For example, children should be able to understand stories, appropriately use oral language, recognize and write the letters of the alphabet, manipulate the sounds of language, write short words using letters or groups of letters from other known words, coordinate their body movements within a sequence of action, show creativity with different

art forms, know number words up to 30, know different geometrical forms, be able to manipulate numerosities up to 10, and know animals, plants as well as human body parts. The curriculum also aims to build children's sensitivity to moral experiences, develop their ability to identify and verbally express emotions and feelings, and encourage them to compare and differentiate their points of view and those of others. Thus, a central objective of the French preschool curriculum is to prepare children to live and work among each other's.

Because conventional classrooms may also be subject to influence from Montessori education (e.g., Montessori-inspired materials can be found in conventional classrooms in France), Courtier et al. (2021) also used the scale described above in the conventional classrooms to assess their degree of Montessori implementation. These classrooms had scores that were very low compared to Montessori classrooms.

Description of the French conventional public elementary school curriculum

The French public elementary school curriculum is highly structured and teacher-centered (https://www.education.gouv.fr/programmes-et-horaires-l-ecole-elementaire-9011). It includes as core domains French language and literature, mathematics, science, history, geography, and physical education. Additionally, the curriculum incorporates arts, music, and moral and civic education. Structured into five years, from CP (Cours Préparatoire) to CM2 (Cours Moyen 2), the curriculum progressively builds on knowledge and skills. Teachers play a central role in delivering content and guiding student learning through a predetermined syllabus, ensuring consistency and adherence to national standards. Qualitative and quantitative regular assessments and evaluations are employed to monitor and support students' progress.

Complementary analyses

We ran a series of complementary frequentist analyses to assess the robustness of the findings. First, a Yuen's test on 20% trimmed means confirmed a difference between the Montessori and the Conventional groups in terms of math problem-solving scores ($t(58.97)=2.05$, $p=.044$, $d = 0.34$ [95% CI: 0.10, 0.63]). This difference remained significant using a robust M-estimator approach ($p = .013$). Second, the difference between groups in terms of math problem-solving remained significant when controlling for a number of covariates, including math problem-solving performance in pre-K ($\beta=.49$, $p=.024$), vocabulary in pre-K ($\beta=.54$, $p=.020$), reading performance in pre-K ($\beta=.57$, $p=.013$), age at testing ($\beta=.52$, $p=.011$), and sex ($\beta=.54$, $p=.008$). Third, although the vast majority of children in the Montessori group followed the three years of the Montessori early childhood curriculum because they enrolled at age 3 (n=29), ten children enrolled later and did not follow the complete curriculum. To examine whether the inclusion of these participants affected our findings, we repeated our analyses excluding their data. As can be seen in **Supplementary Table S5**, this did not change any of our results. Specifically, the difference between the Montessori and the Conventional groups remained significant for math problem solving ($t(85)=3.17$, $p=.002$, $d = 0.64$), but it was not significant for reading fluency ($t(85)=0.37$, $p=.709$, $d = 0.09$). Therefore, our findings remain robust to the exclusion of outliers, inclusion of covariates, and exclusion of the few children with an incomplete Montessori early childhood curriculum.

Supplementary Table S1. Descriptions of tests.

Skill	Test	Description	Scoring
Reading fluency[1]	Evaluation du langage oral et écrit 6-12 (reading fluency subtests) (Launay, L., Maeder, C., Roustit, J., & Touzin, M. (2018). Évaléo 6-15. Ortho edition)	Read out loud two 450-word texts as quickly and as accurately as possible within 2 minutes. One text was sensical while the other was nonsensical.	Number of words correctly read on average for both texts (0-450).
Math problem-solving[1]	Woodcock-Johnson III (Applied Problems subtest) (Woodcock, Mather, McGrew, & Wendling, 2001).	Respond to 63 increasingly difficult math problems assessing conceptual understanding. The first items involved clock reading and calculating with coins. Items then progress onto word problems requiring understanding of base-10, place-value, and multi-step reasoning. The test is stopped after participants made six consecutive errors, irrespective of page boundaries (consistent with Courtier et al., 2021). The test was administered from item 15 onwards to capture the full range of math problem-solving abilities in our sample.	Number of correctly completed items (0-63).
Arithmetic fluency[2]	Woodcock-Johnson III (Math Fluency subtest) (Woodcock, Mather, McGrew, & Wendling, 2001).	Solve as many arithmetic operations (addition, subtraction, and multiplication) as possible within 3 minutes. This test measures computational speed and procedural efficiency rather than conceptual understanding.	Number of correctly solved operations (0-160).
Short-term and working memory[1]	Corsi Block Tapping task (Corsi, 1972)	Repeat a spatial sequence shown by the experimenter by touching blocks glued onto a wooden board. The sequence progressively increases in difficulty, from 2 to 9 blocks[3]. The sequence is first repeated in the same order as the experimenter, then in reverse order. For each sequence, there are four chances to succeed.	Maximum number of blocks correctly repeated forward (score=0-9) for short-term memory and maximum number of blocks correctly repeated backward for working memory (score=0-9).
Sharing[1]	Dictator Game (Benenson et al., 2007)	Share candies with other children. Children first choose their 10 favorite candies among a choice of 30. They are then told that, unfortunately, not enough candies are available for other children. But participants could donate any number of their candies to other children (the choice is made while the experimenter is looking away).	Number of candies given (0-10).
Theory of Mind (empathy)[2]	Story Based Empathy Task (Dodich et al., 2015)	Complete a 3-panel comic strip with a fourth panel out of three possibilities. Three different categories of comic strips were presented: stories involving the identification of intentions (6 items), stories involving the identification of emotional states (6 items), and stories that do	Number of correctly completed stories (0-12)[4].

		not involve identification of either intentions or emotions (control items, 6 items).	
Theory of Mind (emotion)[2]	Reading the Mind in the Eyes (Baron-Cohen et al., 2001)	Select out of four propositions the word that best describes what a person in a photograph is feeling or thinking. Photographs were black-and-white and depicted the eye region of a different individual (28 items).	Number of correctly recognized emotion or thought (0-28).

Notes. [1], Test administered individually. [2], Test administered in a whole-class setting. [3], The maximum number of blocks shown in the backward condition was only six for the first cohort, in line with Courtier et al. (2021). However, because we noticed that some children (n=7) were at ceiling, the maximum number of blocks in the backward condition was increased to nine blocks for the other cohorts. Results do not change after excluding these children. [4], Pre-registered scores for this task ranged from 0 to 18 including the control task. In the end, we opted for a theory of mind score with the two tasks of attribution of intention and emotion (score from 0 to 12) and regressed out success on the control items.

Supplementary Table S2. Sample characteristics at pre-K (age 4) for the participants on which follow-up data is available.

	**Montessori**	**Conventional**	**p**	**Cohen's d**
Math [(1)]	10.66	10.36	0.732	0.08
Vocabulary [(2)]	12	12.26	0.651	-0.11
Reading [(3)]	2.34	3.18	0.382	-0.21
Executive functions [(4)]	15.21	16.37	0.751	-0.07
Male/Female	17/12	25/25	0.459	NA
Age (years)	4.66	4.63	0.559	0.14

Notes. [(1)] Applied Problems subtest of the Woodcock-Johnson III battery, [(2)] Evaluation du Langage Oral test, [(3)] Lecture subtest of the Evaluation Des fonctions cognitives et des Apprentissages battery, [(4)] Head Toes Knees Shoulders task.

Supplementary Table S3. End of treatment effect (i.e., scores at the end of kindergarten) for the participants on which follow-up data is available.

	**t**	**df**	**p**	**Cohen's d**	**BF_{10}**	**BF_{01}**
Vocabulary [(2)]	-0.97	94	0.333	-0.20	0.33	**3.02**
Phonological awareness [(3)]	-0.35	94	0.728	-0.07	0.23	**4.35**
Reading [(4)]	3.15 [(1)]	61.78	**0.003**	0.68	**27.98**	0.04
Pragmatic skills [(5)]	-0.35	91	0.729	-0.07	0.23	**4.28**
Math problem solving [(6)]	-0.39	94	0.697	-0.08	0.23	**4.29**
Counting knowledge [(7)]	-0.27	92	0.785	-0.06	0.23	**4.39**
Quantitative knowledge [(8)]	-1.44	92	0.154	-0.30	0.54	1.84
Short-term memory [(9)]	-0.05	94	0.961	-0.01	0.22	**4.58**
Working memory [(9)]	-1.36	93	0.176	-0.28	0.49	2.02
Self-regulation [(10)]	0.68	94	0.498	0.14	0.27	**3.74**
Planning [(11)]	-0.75	56	0.453	-0.20	0.34	2.95
Sharing [(12)]	0.73	95	0.468	0.15	0.27	**3.64**
Distributive justice (overall) [(13)]	0.16	95	0.875	0.03	0.22	**4.55**
Distributive justice (resources) [(13)]	-0.46	95	0.645	-0.10	0.24	**4.19**
Distributive justice (common good) [(13)]	1.52	95	0.132	0.31	0.60	1.67
Distributive justice (power) [(13)]	-0.78[(1)]	93.67	0.438	-0.16	0.28	**3.62**
Social competence (justice) [(14)]	-0.85	95	0.399	-0.18	0.30	**3.35**
Social competence (flexibility) [(14)]	-1.82	95	0.071	-0.38	0.93	1.07
Theory of mind [(15)]	0.37	93	0.716	0.08	0.23	**4.31**

Note. For frequentist statistics, the Bonferroni-corrected significance threshold was $p < .003$. Significant results are in bold. Cohen's ds represent effect sizes that can be considered small (.05), medium (.05 to <.20) or large (≥.20) in the context of an education intervention (Kraft, 2020). Bayesian statistics: BFs > 3, which correspond to at least substantial evidence for either the null (BF_{01}) or the alternative (BF_{10}) hypothesis, are in bold. [(1)] A Welsh correction was applied for this test because the condition of equality of variances was

violated, [2] Evaluation du Langage Oral test, [3] Phonologie subtest of the Evaluation Des fonctions cognitives et des Apprentissages battery, [4] Lecture subtest of the Evaluation Des fonctions cognitives et des Apprentissages battery, [5] Scalar task, [6] Applied Problems subtest of the Woodcock-Johnson III battery, [7] Counting task, [8] Tokens task, [9] Corsi Block Tapping task, [10] Head Toes Knees Shoulders task, [11] Planification subtest of the Evaluation Des fonctions cognitives et des Apprentissages battery, [12] Dictator Game, [13] Resource allocation task, [14] Social Problem Solving Task-Revised, [15] Wellman and Liu (2004) task.

Supplementary Table S4. Descriptive statistics.

	Pedagogy	N	Missing	Mean	Median	SD	Min	Max
Reading fluency	Conventional	58	0	221.63	213.50	62.15	83.50	395.50
	Montessori	39	0	217.45	220	58.93	90	318.50
Math problem solving	Conventional	58	0	34.55	35	4.56	22	43
	Montessori	39	0	36.72	36	2.67	30	42
Arithmetic fluency	Conventional	51	7	63.14	62	19.46	13	104
	Montessori	39	0	62.62	60	15.94	34	102
Short-term memory	Conventional	58	0	6	6	0.90	3	8
	Montessori	39	0	6.03	6	0.67	5	8
Working memory	Conventional	58	0	5.29	5	0.70	3	6
	Montessori	39	0	5.21	5	0.77	3	6
Sharing	Conventional	58	0	3.28	3.50	1.55	0	6
	Montessori	39	0	3.03	3	2.19	0	10
Theory of mind (empathy)	Conventional	52	6	9.46	10	2.33	3	12
	Montessori	39	0	8.72	9	2.68	3	12
Theory of Mind (emotion)	Conventional	52	6	17.37	18	3	10	23
	Montessori	39	0	18.95	19	3.20	12	26

Note. N varies because some children were absent for some of the tests (arithmetic fluency and theory of mind).

Supplementary Table S5. Frequentist and Bayesian independent sample t-tests for the children who followed the 3 years of the Montessori early childhood curriculum.

	t	df	p	Cohen's d	BF_{10}	BF_{01}
Reading fluency	0.37	85	0.709	0.09	0.25	**3.99**
Math problem-solving	3.17[1]	85	**0.002**	0.64	**3.92**	0.26
Arithmetic fluency	0.03	78	0.974	0.01	0.24	**4.16**
Short-term memory	-0.36	85	0.719	-0.08	0.25	**4.01**
Working memory	-0.93	85	0.354	-0.21	0.34	2.91
Sharing	-1.25[1]	46.50	0.218	-0.29	0.51	1.94
Theory of mind (empathy)	-1.51	79	0.136	-0.35	0.63	1.58
Theory of mind (emotion)	2.53	79	0.013	0.59	**3.61**	0.28

Note. [1]A Welsh correction was applied for this test because the condition of equality of variances was violated. For frequentist statistics, the Bonferroni-corrected significance threshold was $p < .006$. Significant results are in bold. Cohen's ds represent effect sizes that can be considered small (.05), medium (.05 to <.20) or large (≥.20) in the context of an education intervention (Kraft, 2020). Degrees of freedom (df) vary because some children were absent for some of the tests. Bayesian statistics: BFs > 3, which correspond to at least substantial evidence for either the null (BF_{01}) or the alternative (BF_{10}) hypothesis, are in bold.

References

Courtier, P., Gardes, M., Van der Henst, J., Noveck, I. A., Croset, M., Epinat-Duclos, J., Léone, J., & Prado, J. (2021). Effects of Montessori Education on the Academic, Cognitive, and Social Development of Disadvantaged Preschoolers : A Randomized Controlled Study in the French Public-School System. *Child Development*, *92*(5), 2069-2088. https://doi.org/10.1111/cdev.13575
